# Effect of multiple counselling contacts along the continuum of care on use of postpartum family planning in a cohort of Ethiopian women: a dose-response analysis

**DOI:** 10.1136/bmjopen-2024-084247

**Published:** 2024-12-20

**Authors:** Anne Pfitzer, Gebi Husein Jima, Deborah Sitrin, Firew Ayalew, Saifuddin Ahmed

**Affiliations:** 1 MOMENTUM Country and Global Leadership, Jhpiego Corporation, Baltimore, Maryland, USA; 2 Department of Public Health, College of Health Sciences, Arsi University, Asella, Oromia, Ethiopia; 3 Department of Health Sciences, Global Health, University of Groningen, Groningen, Netherlands; 4 Global Program and Technical Excellence, Jhpiego Corporation, Baltimore, Maryland, USA; 5 Jhpiego Ethiopia, Addis Ababa, Ethiopia; 6 Population, Family and Reproductive Health, Johns Hopkins Bloomberg School of Public Health, Baltimore, Maryland, USA

**Keywords:** Postpartum Women, PUBLIC HEALTH, Ethiopia, Health Services, Organisation of health services

## Abstract

**Objective:**

Dose-response analysis of the effect of each additional contact where family planning (FP) was discussed during antenatal, delivery, postnatal or immunisation visits on the uptake of postpartum family planning (PPFP) within 12 months.

**Design:**

A cohort where pregnant women were enrolled and reinterviewed approximately 12 months postpartum. Life table analyses examined differentials in probabilities of adopting contraception over 12 months postpartum by level of exposure to FP counselling. Competing risks regression analysis examined the dose effects in HRs by the number of maternal, newborn or child health (MNCH) contacts where FP was discussed, adjusted for confounding covariates.

**Setting:**

Two Arsi zone woredas: Oromia and Ethiopia.

**Participants and measures:**

722 pregnant women enrolled, and 706 successfully reinterviewed 12 months postpartum about each MNCH contact during pregnancy, delivery and later visits, whether these included any PPFP counselling and PPFP use.

**Main results:**

Two-thirds of the cohort delivered at home. The average number of MNCH contacts women received was 7.6, while the average number where FP was discussed was 2.8. The cumulative probability of PPFP uptake was higher for women who received FP information during at least one MNCH contact, regardless of place of delivery. Each additional MNCH contact where FP was discussed increased the likelihood of PPFP uptake by 14% (95% CI 8% to 20%) or 9% (95% CI 5% to 13%), depending on place of birth. PPFP did not increase with additional contacts without FP information.

**Conclusions:**

While PPFP conversations immediately after a facility birth generated the greatest chance of affecting use, integrating at every visit in the continuum had more impact.

**Trial registration number:**

ClinicalTrials.gov, NCT03585361.

**STRENGTHS AND LIMITATIONS OF THIS STUDY**

- This is a comprehensive study to provide empirical evidence of integrating family planning (FP) counselling with each maternal, newborn or child health (MNCH) contact as suggested in a ‘continuum of care model’ using household surveys.
- We also isolated the effect of FP discussions from any effect or selection bias from contact with the health system by including the number of contacts where FP was not discussed in our models.
- The analysis relies on women’s recall of the timing of contraception adoption or the number of contacts, with the possibility that users of FP had higher recall than non-users.
- Under-reporting of FP counselling, in contrast, may attenuate the effects of counselling.
- We did not analyse the timing of contacts in relation to postpartum family planning (PPFP) adoption, so some women may have started contraception before receiving some of the contacts included in the analysis. While FP information received during antenatal contacts and immediately after delivery can be presumed to have preceded PPFP adoption, and while it is likely that postnatal contacts and early immunisation contacts also preceded PPFP adoption, it is unknown if PPFP adoption preceded or followed immunisation contacts that occurred after the initial few weeks of life.

## Introduction

 Family planning (FP) programmes contribute to reducing high-risk births, including those that occur at short intervals.^1^ Evidence reviews on postpartum family planning (PPFP) interventions and strategies specifically have shown mixed, but often positive, impacts on PPFP uptake for integrating counselling and services within antenatal care (ANC), predischarge maternity and postnatal care (PNC), as well as with immunisation and other services for young children.^2^,^4^ Many review articles^2^,^4^ lacked details of information on how the PPFP services were available to women during contacts at birth or in the postnatal and extended postpartum period. Cleland *et al*’s^2^ review of 35 studies concluded that a single antenatal counselling session lacked impact on increasing PPFP use but had significant effects only with multiple counselling sessions. In that review, some studies looked at the effect of intervening at just one phase in the continuum from pregnancy to delivery care to the initial and extended postnatal periods, while other studies investigated the impact of intervening at multiple phases. The study concluded that ‘the ideal strategy for improving PPFP is to incorporate contraceptive advice and services across the continuum of reproductive healthcare.’

The WHO has endorsed integration along the entire continuum of maternal, newborn and child health (MNCH) care and published Programming Strategies for Postpartum Family Planning in 2013 to guide countries through thinking of all the strategies that could be employed during pregnancy, the time of birth and up to 12 months postpartum to increase the use of PPFP.^5^ Policies and programmes lag behind in implementing PPFP at scale, but the FP2020 movement embraced PPFP as an intervention which has since been reaffirmed under FP2030.^6^ Opportunities for integrating PPFP may increase in number as the WHO issued recommendations for eight antenatal contacts, instead of four, although encouraging countries to explore a mix of facility and home or community contacts.^7^ The WHO also recommended a minimum of three postnatal contacts in addition to a predischarge or home assessment within 24 hours of birth,^8^ although achieving high rates of coverage for these contacts remains a daunting challenge.^9^ Countries determine their own immunisation schedules depending on the local epidemiological context; nevertheless, most countries administer three doses of diphtheria, pertussis and tetanus (often combined with other vaccines) and a measles vaccine in infancy. Immunisation coverage rates are among the highest of all contacts along the continuum of care.^5^ Thus, if woman-baby pairs receive all recommended contacts along the continuum of care, this results in a dozen or more interactions between a mother and health worker, whether in facility or community settings. Each of these contacts could potentially fully integrate a discussion of the woman’s FP needs and options for PPFP (and possibly involve their partners too). The current global evidence is not available for the cumulative benefit of each additional contact where FP is discussed on her eventual adoption of the practice. Nor has the effect on individual women of hearing about FP at all contacts she has with the healthcare system before and after a birth been quantified in terms of the probability of use of PPFP by 12 months postpartum.

An analysis of interpregnancy intervals from Demographic and Health Surveys (DHS) found that 47% of non-first births in Ethiopia occurred sooner than the expert-recommended 24 months.^10 11^ More recently, the proportion of women using modern FP increased substantially from 24% in 2005 to 41% in 2019 and those who gave birth at health facilities have also increased from 5% to 48% during the same period.^12^

A research team from Jhpiego and Arsi University collected data under a study entitled ‘Utilising All Health System Contact to Offer Postpartum Family Planning in Ethiopia’, which strengthened the integration of FP along the continuum from pregnancy to the extended postpartum at the primary healthcare level, which in Ethiopia includes health centres, health posts and home visits. A longitudinal survey conducted in Ethiopia concurrently with this study suggested that only postnatal PPFP counselling influenced uptake.^13^ This paper presents a dose-response analysis to examine the effect on the uptake of PPFP up to 12 months postpartum of each additional antenatal, delivery, postnatal or child immunisation contact where FP was discussed.

## Methods

### Study setting, design, sampling and interventions

The study setting, design, sampling and intervention overview have been published previously.^14^ In summary, the study was conducted in the Arsi zone, Oromia Region of Ethiopia, in 2017–2018. Eight health centres in two adjacent *woredas* (districts) received technical support to initiate PPFP counselling during ANC, predischarge from labour and delivery, PNC and immunisation visits. Labour and delivery providers also received separate training in the insertion of postpartum intrauterine devices (IUD). All health centres received follow-up supportive supervision to encourage systematic integration of PPFP into MNCH visits along the continuum of care from pregnancy up to 12 months postpartum. Two primary healthcare units (defined as a health centre with health posts in that centre’s catchment areas) were randomly selected to receive additional inputs to initiate PPFP counselling and messages at health posts and during home visits to pregnant and postpartum women (intervention arm). Health posts in the community intervention area also received supportive supervision to promote systematic integration of PPFP, whereby health extension workers (HEWs) who staff the health posts were encouraged to use a modified Integrated Maternal and Child Health Card with nudges to remind them to counsel women at key contacts, as described in a separate publication.^15^ HEWs in the community intervention areas were also instructed to engage women development army volunteers to identify pregnant and postpartum women and promote the utilisation of services at health posts and health centres. Women living in areas receiving community intervention in addition to health centre interventions were considered the intervention arm and women living in areas receiving only health centre interventions were considered the comparison arm.

The study sample size was calculated to identify differences in PPFP uptake by study arms to examine the effect of the add-on community interventions^14^; the minimum sample required for that analysis was 750 women (375 per arm).^14^

Using information from HEWs and community guides, we visited 772 pregnant women in their homes, enrolled them in the study and prospectively followed them approximately 1 year postpartum. These women were enrolled during their second and third trimesters and first interviewed in February–March 2017, and 706 of them were successfully followed up and re-interviewed in May 2018 (9% loss to follow-up). The interview questionnaire is available online in the Open Science Framework repository at DOI: 10.17605/OSF.IO/9EC6V.^16^ All participating women resided in areas served by health centres that received technical support to integrate PPFP into other services; 351 women (49.7%) lived in areas that also received additional community PPFP interventions (intervention arm). For the purpose of the ‘dose-response’ analysis presented in this paper, all women enrolled in the study were viewed as a single cohort with varying exposure to PPFP interventions. Our analysis adjusted for the intervention arm differentials.

### Patient and public involvement

Before the study began, a project expert in social and behavioural change conducted a rapid qualitative assessment in a nearby woreda. Her conclusions related to local perceptions of contraceptive implants leading to greater pains during the hungry seasons led us to select different study woredas out of concern of limiting the generalisability of our study, although the effects of these beliefs are more likely to affect method choice than whether to use PPFP. Other than pretesting interview tools with local women visiting a health facility, no further patient or public involvement occurred until the end of the study, when HEWs from the study woredas were involved in national dissemination activities.

### Data collection procedures

The interviews, conducted in women’s homes, probed how many and what health contacts they had (provider, type of service delivery point and, in the case of health centres the exact facility) from the beginning of pregnancy up to the time of the interview. Women also reported on their use of PPFP, including the timing of initiating a method and method switching since birth.

### Data analysis

#### Overview of analyses

This paper analyses data from the final interviews conducted in May 2018, at which point the women were 10–16 months postpartum. First, we examined the data quality and performed exploratory data analysis (checked frequencies, mean, percentage, median, outliers and missing values). Second, we conducted a life table analysis to examine the differences in the adoption probabilities of a contraceptive method over 12 months after delivery by the level of exposure to FP counselling during MNCH contacts. Finally, we conducted a multivariate analysis to adjust for the effect of potential confounding covariates on PPFP adoption hazard rates. We used a competing-risk regression model^17^ to calculate the hazards of PPFP adoption at each time point because 43 women (6%) had a subsequent pregnancy during the 1-year postpartum period, which precluded them from adopting PPFP from that point. Pregnancy was considered as the competing risk for not using PPFP in the analysis. The dataset for this study has been shared on United States Agency for International Development Data Library.^18^

#### Defining the ‘dose’

For conducting the dose-response analysis, the intervention ‘dose’ was defined as the total number of antenatal, delivery, postnatal and child immunisation contacts (collectively, we will refer to these contacts as maternal child health or MNCH contacts) where the woman reported a provider talked to her about FP. Women were asked separately about each type of MNCH contact and each point of service (hospital, private facility, health centre, health post and home). For example, women were asked, ‘At how many antenatal visits to a health centre did a staff member talk to you about family planning?’. A similar question was asked for antenatal contacts at hospitals, private facilities, health centres, health posts, home visits by HEWs and home visits by so called ‘Women Development Army’ volunteers. The same set of questions was asked for postnatal contacts. Women who delivered in a facility were asked if someone talked to them about FP prior to discharge. Women were asked about the number of times they received FP information during child immunisation visits to health centres and to health posts/outreach. Immunisations rarely occur at hospitals, nor do they ever occur in private facilities or during home visits, so we did not ask about those to limit the length of the questionnaire. Thus, calculating the ‘dose’ involved summing responses to 14 questions for women who delivered at home and 15 questions for women who delivered at a facility. We also calculated the ‘dose’ for each type of contact: pregnancy, PNC and immunisation contacts. Women who delivered at a facility were eligible for an additional contact, so all analyses were done separately for women who delivered at home versus at a facility.

#### Contacts without FP content

We then calculated the number of MNCH contacts where FP was *not* discussed by subtracting the number of MNCH contacts where FP *was* discussed from the total number of MNCH contacts. The total number of contacts was calculated by summing responses to 14 questions for women who delivered at home and to 15 questions for women who delivered in a facility. Again, separate questions were asked on the number of contacts of different types (antenatal, delivery, postnatal and child immunisation) and points of service (hospital, private facility, health centre, health post and home). These questions preceded the questions used to calculate the intervention ‘dose’. For example, ‘How many times did you receive antenatal care at a health centre?’ preceded ‘At how many antenatal visits to a health centre did a staff member talk to you about family planning?’. Again, we calculated the total number of MNCH contacts where FP was not discussed as well as the number of each type of contact where FP was not discussed.

#### Life table analysis of FP use or subsequent pregnancy

In order to measure the effect of repeat doses of FP discussions over multiple visits on a postpartum FP use outcome and because not all women adopt a method and some may experience pregnancy before they do, we conducted a life table analysis and fitted a competing risk survival model using Stata (Release 14).^19 20^ Because a subsequent pregnancy during the postpartum period may alter the needs of contraceptive use, we use the pregnancy incidence as a competing risk in the hazards regression model. A competing risk model correctly estimates the marginal probability of an event in the presence of competing events, which is referred to as the cumulative incidence function (CIF). Time from delivery to PPFP uptake was based on women’s reports of how long after delivery they started using PPFP. We included all modern contraception methods (IUDs, implants, pills, injectables, sterilisation and condoms) except lactational amenorrhoea (LAM), which we did not include in this study’s definition of PPFP because it cannot be used past 6 months postpartum and LAM use does not exclude women from adopting another contraceptive method. Only eight women reported using LAM, of whom four adopted another PPFP method. For women with a subsequent pregnancy, we estimated the time between index birth and subsequent pregnancy by calculating the time between index birth and final interview, then subtracting the number of months pregnant at the final interview. For women who already had a subsequent birth, we subtracted the time between the date of the subsequent birth and the final interview, then subtracted 9 months to account for the pregnancy duration. For five women, the estimated start of the subsequent pregnancy was less than 1 month after the index birth (because the subsequent pregnancy was less than 9 months or dates were incorrectly reported), so we coded the pregnancy as occurring at 1 month postpartum. We compared the CIF for women who reported at least one MNCH contact where FP was discussed and women who reported no FP discussions; analysis was stratified by place of delivery (home vs facility).

#### Multivariate analysis of the cumulative effect of doses on PPFP use

To calculate the cumulative effect of each MNCH contact on postpartum contraceptive uptake, we fitted multivariate competing-risks regression models using the method developed by Fine and Gray to estimate the adjusted sub-HR (aSHR) and 95% CI.^21^ Time from delivery to PPFP uptake or pregnancy was the dependent variable. The observation period was limited to the first 12 months postpartum; all at-risk observations (women who had not adopted contraception by 1 year postpartum) were censored at 12 months. Women interviewed prior to reaching 12 months postpartum who did not adopt a modern contraceptive method by the time of the interview were censored at the month of the interview.

The main covariate of interest is the number of MNCH contacts where FP was discussed. Models also include the number of MNCH contacts where FP was not discussed to separate the effect of receiving information on FP from the effect of having contact with the health system. Only 15 women (2%) reported more than 12 contacts where FP was discussed, and 28 women (4%) reported more than 12 contacts where FP was not discussed. We capped the highest values to 12, considering very few cases at the higher values.

Models controlled for a woman’s age (20 or younger, 21–25, 26–30, 31–35 and 36+), marital status (married vs not married), education (none, at least some primary and at least some secondary), religion (Ethiopian Orthodox, Muslim or other) and the national wealth quintile of the household where she resides. Household placement within national wealth quintiles was determined using the Equity Tool (equitytool.com), which calculates wealth status by comparing responses to a series of questions to national data from the most recent DHS.

#### Additional multivariate analyses by type of MNCH contact

We also fitted separate regression models to examine the effects of each type of MNCH contact. Models for pregnancy, postnatal and immunisation contacts included the number of respective contacts with and without FP discussions, plus the same covariates used for the main model. We capped each variable at six contacts due to the small number that reported more than that. Receipt of predischarge FP information was a binary variable, and we only fit this model for women who delivered in a facility since women who delivered at home were ineligible by definition.

#### Modelled estimates of PPFP use if FP were discussed across eight MNCH contacts

Lastly, for better interpretation of the results for policy relevance, we used our regression model estimates to extrapolate (predict) the FP use probabilities over the postpartum period under three scenarios: all women had zero MNCH contacts with the health system; all women had eight MNCH contacts with the health system, but FP was never discussed; and all women had eight MNCH contacts and FP was discussed at all of them. We selected eight contacts as a realistically achievable target under the current level of MNCH care if women receive at least four antenatal contacts, deliver in a facility, receive at least one postdischarge postnatal contact and their children have two immunisation visits during the first year of life. We plotted the cumulative incidence function with these three scenarios to visualise the effect of using all contacts to discuss FP.

## Results

Of 706 women completing the postpartum interview, 429 (61%) had given birth at home and 276 (39%) in a facility (one woman missing the place of delivery was excluded from analysis). A detailed profile of the study participants is presented in detail in a separate publication.^14^

**Figure 1:**
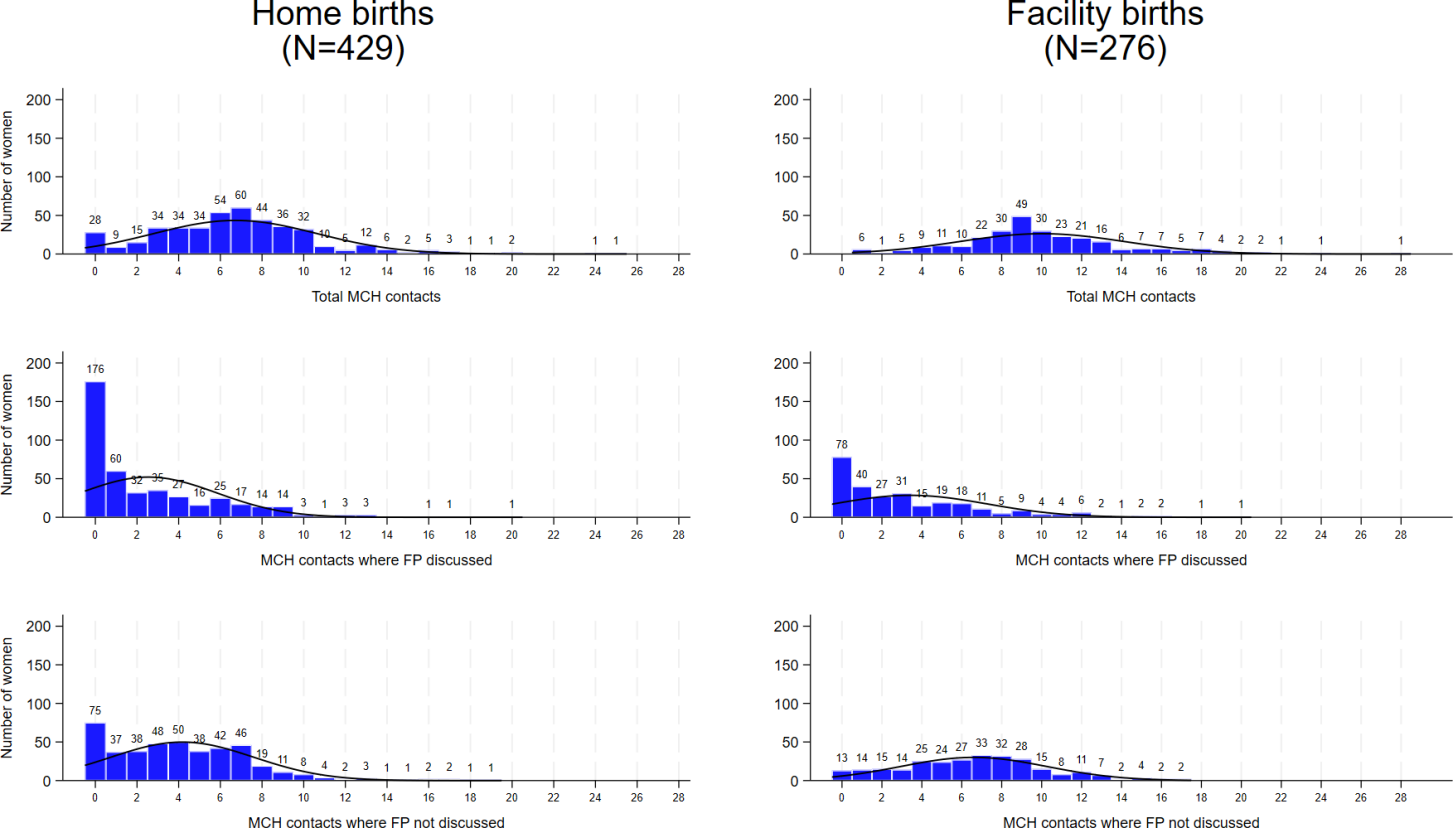
Distribution of the number of MNCH contacts. FP, family planning; MNCH, maternal, newborn or child health.

The average number of MNCH contacts women received was 7.6, while the average number of MNCH contacts where FP was discussed was only 2.8. The distribution of total MNCH contacts received was approximately normal for both women who delivered at home and at a facility (figure 1) with some right skewing because a small number of women reported more than 16 contacts. On the other end, 28 women who delivered at home (6.5%) reported no contact with the health system in connection with their index birth. The median number of MNCH contacts was seven for women who delivered at home and nine for women who delivered at a facility.

The distribution of MNCH contacts where FP was discussed was heavily skewed; 176 women who delivered at home (41%) and 78 who delivered at a facility (28%) reported never discussing FP during MNCH contacts within the health system. The median number of MNCH contacts where FP was discussed was one for women who delivered at home and two for women who delivered at a facility. The distribution of MNCH contacts where FP was not discussed was also skewed for women who delivered at home while approximately normal for women who delivered at a facility.

### Effect of FP discussions during MNCH contacts

**Figure 2:**
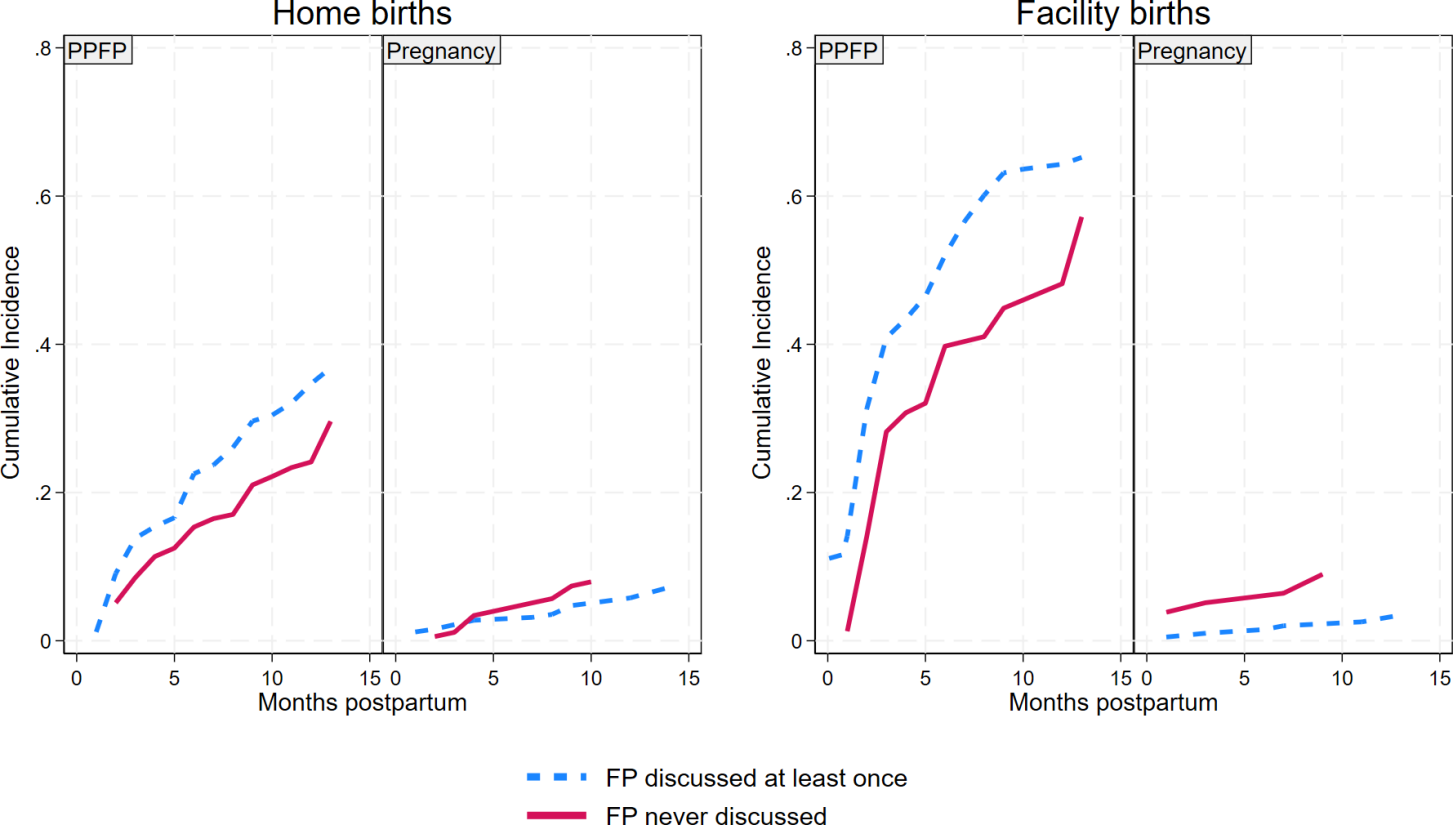
Cumulative incidence of either PPFP use or pregnancy. FP, family planning; PPFP, postpartum family planning.

Life table analysis (figure 2) showed the cumulative probability of PPFP uptake was higher for women who received FP information during at least one MNCH contact than for women who did not, regardless of place of delivery. At 12 months postpartum, the cumulative probability of PPFP uptake for women who delivered at home and at a facility was 35% and 64%, respectively, if they received FP information. It was only 24% and 48%, respectively, if they did not receive FP information. (Complete life tables are provided as online supplemental file 1.) Conversely, pregnancy incidence was lower for women who received FP information than those who did not, regardless of place of delivery.

Our regression models (table 1) show the aSHR of PPFP uptake increased by 14% (95% CI 1.08 to 1.20; p<0.001) with each additional MNCH contact that included a discussion of FP for women who delivered at home and 9% (95% CI 1.05 to 1.13; p<0.001) for women who delivered in a facility. PPFP did not increase significantly with additional MNCH contacts where FP was not discussed (aSHR=1.06, 95% CI 0.99 to 1.13; p=0.104 for home births, aSHR=1.01, 95% CI 0.95 to 1.07; p=0.743 for facility births).

**Table 1:** The aSHR of adoption of modern contraception over the first year postpartum with MNCH contacts with and without FP counselling

	Home births			Facility births		
	aSHR	P value	(95% CI)	aSHR	P value	(95% CI)
MNCH contacts						
MNCH contact with FP discussion	1.14	<0.001	(1.08 to 1.20)	1.09	<0.001	(1.05 to 1.13)
MNCH contact without FP discussion	1.06	0.104	(0.99 to 1.13)	1.01	0.743	(0.95 to 1.07)
Age of woman						
≤20	0.63	0.291	(0.27 to 1.48)	1.02	0.921	(0.65 to 1.61)
21–25	Reference group					
26–30	1.27	0.264	(0.84 to 1.92)	0.76	0.133	(0.53 to 1.09)
31–35	0.96	0.913	(0.49 to 1.90)	0.55	0.100	(0.27 to 1.12)
36+	0.87	0.734	(0.38 to 1.98)	0.42	0.007	(0.22 to 0.79)
Marital status						
Not married	Reference group					
Married	1.39	0.565	(0.45 to 4.32)	1.63	0.483	(0.41 to 6.44)
Years of education						
None	Reference group					
1–8 years	1.30	0.157	(0.90 to 1.86)	1.38	0.124	(0.92 to 2.06)
9+ years	1.58	0.351	(0.60 to 4.15)	1.99	0.006	(1.22 to 3.24)
Religion						
Orthodox	Reference group					
Muslim	0.29	<0.001	(0.20 to 0.43)	0.62	0.011	(0.43 to 0.90)
Other	1.82	0.176	(0.76 to 4.31)	1.19	0.678	(0.52 to 2.73)
Wealth quintile						
Lowest	Reference group					
Second	0.99	0.975	(0.49 to 1.98)	0.83	0.715	(0.30 to 2.30)
Middle	0.84	0.567	(0.45 to 1.54)	0.86	0.741	(0.36 to 2.06)
Fourth	0.77	0.449	(0.39 to 1.52)	1.23	0.623	(0.54 to 2.82)
Highest	0.86	0.725	(0.38 to 1.95)	1.07	0.883	(0.44 to 2.58)
Live children						
0	Reference group					
1–2	1.40	0.749	(0.18 to 11.15)	1.52	0.503	(0.44 to 5.23)
3–4	1.02	0.984	(0.14 to 7.65)	1.66	0.425	(0.48 to 5.82)
5+	0.61	0.627	(0.08 to 4.51)	1.00	0.997	(0.26 to 3.85)
Study arm						
Comparison arm	Reference group					
Intervention arm	1.23	0.295	(0.84 to 1.80)	0.82	0.224	(0.59 to 1.13)

FP discussion distributions during each type of MNCH visit.

aSHRadjusted sub-HRFPfamily planningMNCHmaternal, newborn or child health

**Figure 3:**
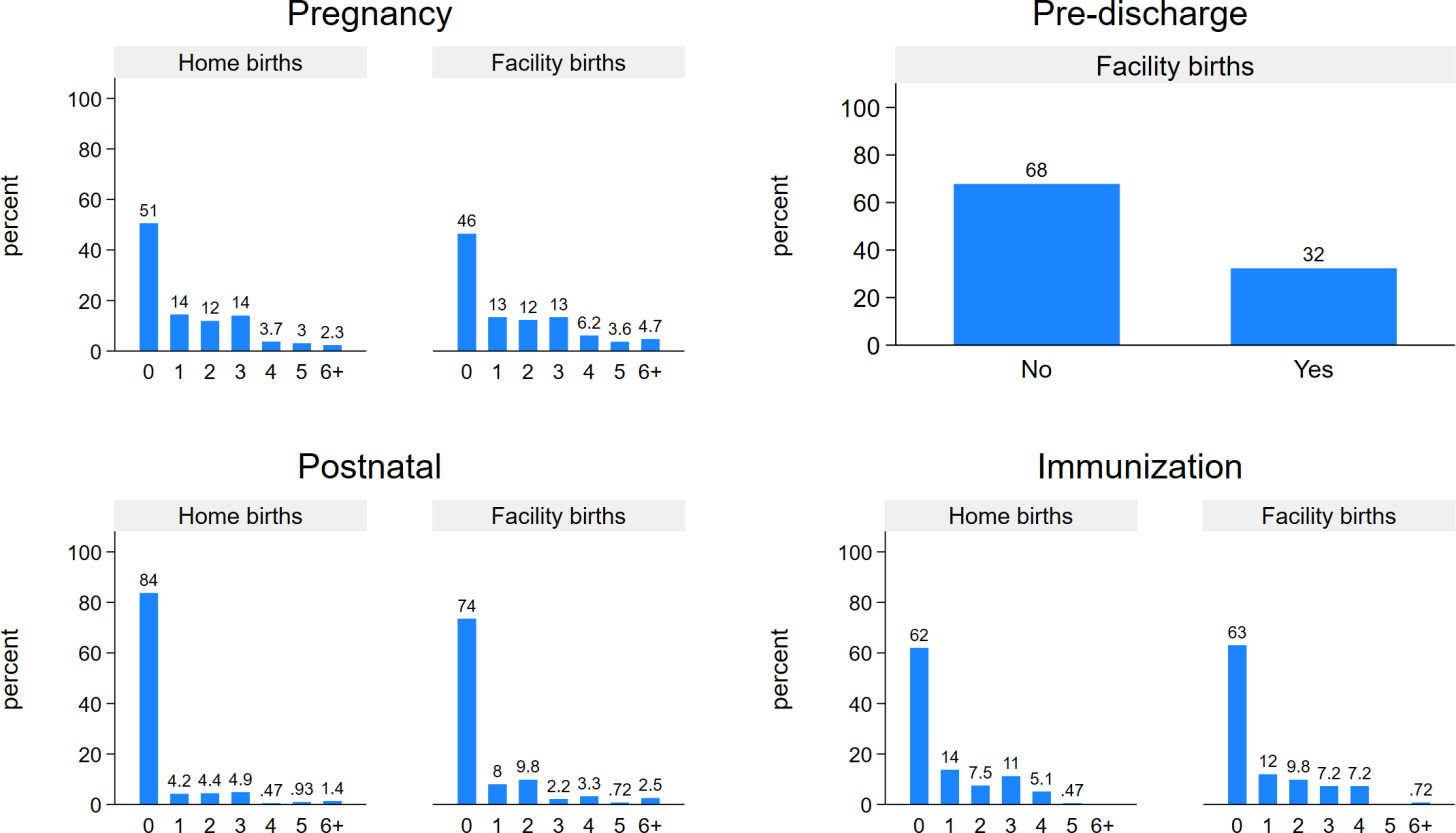
Percent of women who had maternal, newborn or child health contacts with family planning discussion.

The proportion of women who had at least one pregnancy (ANC) contact where FP was discussed was 49% if delivered at home and 54% if at a facility, at least one postnatal contact where FP was discussed was 16% if delivered at home and 26% if at a facility, and at least one child immunisation visit where FP was discussed was 38% if delivered at home and 24% if at a facility (figure 3). Among women who delivered in a facility, 32% received information on FP before discharge.

The aSHRs from the separate models for each type of MNCH contact are shown in table 2. (Complete models are provided in Annex A and Annex B in online supplemental file 1) Again, the hazards of PPFP uptake increased with additional contacts where FP was discussed. This was true for pregnancy contacts and immunisation contacts, both for women who delivered at home and at a facility, though the hazard increases were even greater for women who delivered at home, with a 30% (95% CI 1.16 to 1.44; p<0.001) increase for each additional pregnancy contact and 36% (95% CI 1.17 to 1.57; p<0.001) for each immunisation contact compared with 14% (95% CI 1.05 to 1.23; p=0.001) and 17% (95% CI 1.01 to 1.36; p=0.04) for women who delivered at a facility. The largest SHR increase was 76% comparing women who delivered at a facility and received predischarge information on FP compared with women who did not receive information before discharge (95% CI 1.32 to 2.35; p<0.001). There were mixed results for postnatal contacts. For women who delivered at home, postnatal contacts *without* FP discussion increased the hazard of PPFP adoption by 13% (95% CI 1.00 to 1.28; p=0.05), while contacts with FP discussion showed a lower increased hazard of 8%, which was not statistically significant (95% CI 0.94 to 1.24; p=0298). For women who delivered at a facility, the hazard of PPFP uptake increased by 15% (95% CI 1.07 to 1.24; p<0.001) with each additional postnatal contact that included a discussion of FP, while the hazard did not increase with additional postnatal visits without FP discussion.

**Table 2:** The aSHR of adoption of modern contraception over the first year postpartum for each type of MNCH contact

	Home births			Facility births		
	aSHR*	P value	(95% CI)	aSHR*	P value	(95% CI)
Model A: pregnancy contacts						
MNCH contact with FP discussion	1.30	<0.001	(1.16 to 1.44)	1.14	0.001	(1.05 to 1.23)
MNCH contact without FP discussion	0.97	0.683	(0.86 to 1.10)	1.05	0.243	(0.97 to 1.15)
Model B: delivery contact						
FP discussed predischarge	Not applicable			1.76	<0.001	(1.32 to 2.35)
FP not discussed predischarge				Reference group		
Model C: postnatal contacts						
MNCH contact with FP discussion	1.08	0.298	(0.94 to 1.24)	1.15	<0.001	(1.07 to 1.24)
MNCH contact without FP discussion	1.13	0.050	(1.00 to 1.28)	0.95	0.296	(0.87 to 1.04)
Model D: immunisation contacts						
MNCH contact with FP discussion	1.36	<0.001	(1.17 to 1.57)	1.17	0.040	(1.01 to 1.36)
MNCH contact without FP discussion	1.18	0.006	(1.05 to 1.33)	1.02	0.776	(0.89 to 1.17)

* All models adjusted for age, marital status, education, religion, wealth and number of live children.

aSHRadjusted sub-HRFPfamily planningMNCHmaternal, newborn or child health

**Figure 4:**
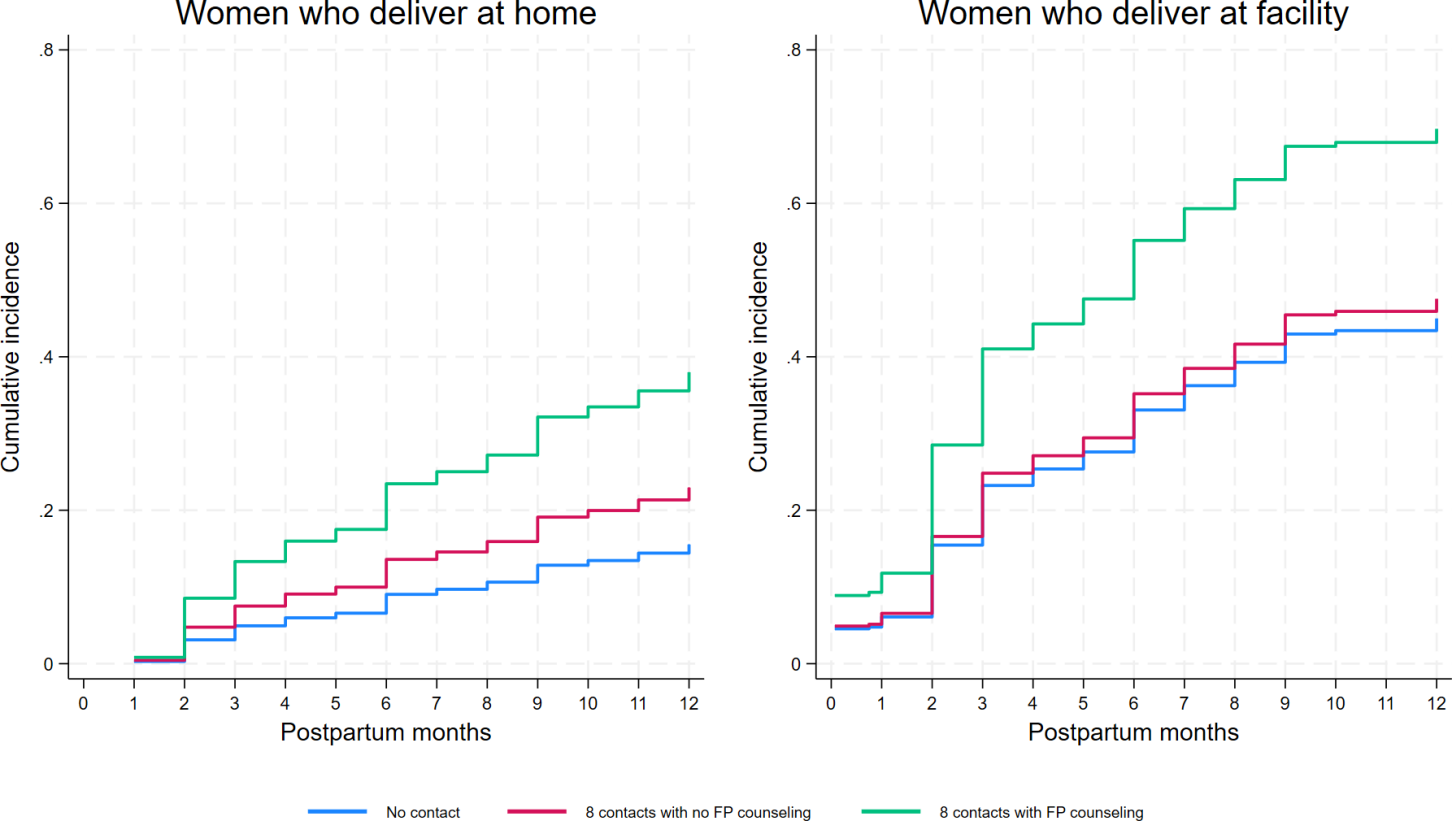
Cumulative incidence curves for three scenarios.

The results from our prediction modelling of three scenarios where all women receive care are shown in figure 4: no contacts with the health system, eight contacts without FP discussions or eight contacts where FP was discussed during all of them. If all women had eight MNCH contacts where FP is discussed, PPFP uptake by 12 months postpartum would be much higher than the other two scenarios and would be approximately 40% for women who deliver at home and over 60% for women who deliver at a facility.

## Discussion

In this cohort of Ethiopian women, our study results show that each additional MNCH contact with PPFP counselling, where FP was specifically discussed, increased the likelihood of PPFP uptake among women who delivered at home or at a health facility, while MNCH contacts where FP was not discussed were associated with small but statistically insignificant increases in PPFP uptake. Because many women have a small number of contacts with the health system and every PPFP-related interaction matters, every MNCH contact with pregnant women during the first year postpartum should be seen as an important opportunity to provide FP information and discuss her options, preferences and needs.

This analysis found the benefits of FP discussions at different points on the continuum from pregnancy through the first year postpartum. The immediate aftermath of childbirth seems particularly opportune for discussing healthy timing and spacing of pregnancies and the use of PPFP for women who deliver within facilities. Women who deliver in facilities may make decisions to adopt PPFP at that time or opt to take up a method at a later time. In this cohort, FP discussions during pregnancy and child immunisation contacts seemed to have an impact, especially for women who delivered at home. Our study suggested FP discussions during PNC were important for women who delivered in facilities but were inconclusive for women who delivered at home. We must acknowledge that our models for the effect of FP discussions at each type of contact did not adjust for FP discussions during other types of contacts, since the number of women who received FP information at just one type of contact was too small. There may be mediating effects across contacts that we were unable to examine. It is conceivable that women who received FP information during pregnancy did not desire or need further FP information during postnatal contacts because they had already made up their minds or had already received a method, and women who did receive FP information during PNC contacts were generally more hesitant to adopt PPFP. Also, few women received postnatal contacts, especially if they delivered at home; women who received postnatal contacts may have unmeasured advantages over women who did not that wash out the effect of discussing FP during those contacts. Furthermore, this study was not designed to examine the specific effect of an early postnatal home visit that includes a discussion of FP and compare to the effect on PPFP outcomes as counselling predischarge from a facility birth, though early postnatal home visits are meant to compensate for the lack of predischarge care.

Despite interventions at health centres (in all areas) and at health posts and community levels (in some areas) to integrate PPFP with maternal and child services, most women only heard about FP at a small number of visits, if at all, even though the median number of MNCH contacts was seven and nine for women who delivered at home and at a facility, respectively. Low levels of integration of PPFP in MNCH services are not unique to Ethiopia.^22^ This points to the need for new strategies to ensure that these conversations occur systematically. At the global level, a measurement committee of the PPFP Community of Practice met over several consultations to develop a consensus around indicators for tracking the adoption of PPFP at or near the time of birth.^23^ Using routine systems to track the integration of PPFP services, where feasible, could encourage more attention on whether services are systematically integrated, especially if reviewed and analysed at the facility level. In predischarge contact during a facility birth, the integration of PPFP requires sufficient length of stay and the provision of postpartum care. A recent analysis of DHS data from multiple sub-Saharan African countries has illuminated flaws in assuming that care is automatically given and showed that coverage may be lower than anticipated.^24^ Similarly, a client flow analysis in Kenya and India found that FP counselling is not systematically integrated even where programmes have promoted PPFP.^22^

If all women received FP information at eight contacts, then our data suggests PPFP coverage would be close to 40% for women who delivered at home and 60% for women who delivered at a facility, exceeding the current national modern contraceptive prevalence (41%) among *all* married women,^12^ which tends to be higher than contraception use among postpartum women. It would also well exceed the national modern contraceptive prevalence rate among married women at 12 months postpartum (35%) based on analysis of calendar data from the 2016 Ethiopian DHS.^25^ If systematic integration could achieve eight MNCH contacts with FP discussions across the country, it would drive a continued rapid rise in contraceptive prevalence. The new WHO recommendations for eight antenatal contacts open the door for additional opportunities to reach women with information on FP, including in community settings.^7^ The qualitative component of this study highlighted the experience of service providers engaged in increasing integration.^15^

We analysed the effects of contacts that include a conversation about FP in a novel way. We asked women to recall details of each visit separately, providing data about the effects of FP discussions along the continuum of care. That level of detail contrasts with other studies. Understanding the role of repeated conversations at different stages highlights the importance of applying a continuum of care lens to primary care. While this has already been acknowledged as critical to maternal and newborn survival,^26 27^ our study extends this conclusion to the promotion of PPFP.

Our study is unique in that it provides empirical evidence of integrating FP counselling with each MNCH contact, as suggested in the ‘continuum of care model’.^28^ Our results contribute to a growing body of literature seeking to tease out the effects of PPFP counselling during various health visits. Two small cross-sectional studies explored the role of contacts^29^ and contraceptive counselling during maternity care,^30^ with unclear results. Abera *et al* found an association between ANC and PNC with PPFP use in a cluster population survey of women living in or near Gondar, Ethiopia. Morhe *et al* interviewed women attending well-baby clinics in a facility in Ghana and found that exposure to contraceptive counselling at any time in ANC, postnatal wards of maternity or well-baby clinics was not associated with PPFP use. A more rigorous longitudinal study in the Southern region of Ethiopia^13^ interviewed a cohort of pregnant women at several points in time and found an association between PPFP counselling at ANC and PNC or PNC only and PPFP use, but that study did not see an effect of ANC counselling if not repeated postnatally. In contrast, we saw a sizeable effect from FP information provided during pregnancy, and we know many of these women did not receive follow-up information during PNC due to the low coverage of PNC, though we cannot tease out the effect size for women who received information during pregnancy only. Another study in Nepal that assessed the unmet need for women delivering in six hospitals found that the unmet need for PPFP was significantly lower when women were counselled in the immediate postpartum or during later postnatal visits.^31^ Furthermore, that study did not capture health contacts later than a 6-week PNC visit; yet, there is a body of literature about integrating or linking FP with immunisation visits.^32^,^35^ Integrating services during child immunisation visits is warranted, with care to avoid any negative effects on immunisation coverage. We encouraged health centre staff to facilitate intrafacility referrals between immunisation and FP units or providers similar to what has been described in Liberia,^34^ and our results suggest there is an effect on PPFP uptake, particularly for women who delivered at home. We also found increased PPFP use among women delivering at home who brought their child for immunisation but did not discuss FP but cannot determine if they were acting on counselling earlier in the continuum of care or generally represent more care-seeking behaviour and use of health services. A larger study could further tease out the roles and interactions between repeated contacts along the continuum of care.

Our study has some strengths. Our data comes from household surveys of women who completed a detailed questionnaire about each stage of the reproductive continuum, each type of contact they had with the health systems and whether those contacts included a discussion of FP. Blazer’s review of PPFP evidence^3^ concluded that the main drawback with previous studies was the lack of generalisability since they drew from women delivering, attending ANC or seeking immunisations at a facility. By contrast, our cohort, which was recruited via household survey, included 28 women who never had any formal contact with the health system during either pregnancy or the following year. Those women are invisible in the routine health systems data. Our study also incorporates both community-based and facility-based contacts. We also isolated the effect of FP discussions from any effect or selection bias from contact with the health system by including the number of contacts where FP was not discussed in our models.

Our study also has some limitations. Although studies have shown that women were able to recall discussions on FP and receiving PPFP,^36^ there is no evidence that they can accurately recall the timing of contraception adoption or the number of contacts. We attempted to improve recall on the number of contacts by asking multiple questions tied to points of service, though there has been no validation of these types of questions. It is possible that women who use FP could be more likely to recall FP discussions. Under-reporting of FP counselling, in contrast, may attenuate the effects of counselling. Also, we did not analyse the timing of contacts in relation to PPFP adoption, so women may have started contraception before receiving some of the contacts included in the analysis, so those contacts could not have contributed to the woman’s decision to start PPFP. FP information received during antenatal contacts and immediately after delivery can be presumed to have preceded PPFP adoption. With few women adopting PPFP in the initial months after birth, it is likely that postnatal contacts and early immunisation contacts also preceded PPFP adoption. However, it is unknown if PPFP adoption preceded or followed immunisation contacts that occurred after the initial few weeks of life.

Data were collected retrospectively, so there is a possibility of reverse causality. Women may have received contacts because of starting PPFP. Since the intervention encouraged cross-referrals, it is possible that women who received FP were also encouraged to access child immunisation.

## Conclusions

Our study provides empirical evidence on the dose-response effect of integrated FP discussions along the continuum of care, which has important policy and programmatic implications for Ethiopia and elsewhere for improving maternal and child health. Health workers should systematically and routinely discuss the risks of short birth intervals, plan for birth spacing or limiting and counsel mothers on contraceptive choices for postpartum women during each MNCH contact.

## supplementary material

10.1136/bmjopen-2024-084247 online supplemental file 1

## Data Availability

Data are available upon reasonable request. Data may be obtained from a third party and are not publicly available.
