# Supplementary material for: Effect of multiple counselling contacts along the continuum of care on use of postpartum family planning in a cohort of Ethiopian women: a dose-response analysis
Source: BMJ Open. 2024 Dec 20;14(12):e084247. doi: 10.1136/bmjopen-2024-084247 (PMC11667413; doi:10.1136/bmjopen-2024-084247)
Supplement: online supplemental file 1 [file bmjopen-14-12-s001.docx]

**Supplemental Materials**

**Table S1: List of cumulative incidence function (CIF) in presence of competing risks (life tables)**

| **CIF of PPFP adoption** (stratified by place of delivery and whether woman reported at least 1 health system contact where FP was discussed) | | | | | | | | | | | |
| --- | --- | --- | --- | --- | --- | --- | --- | --- | --- | --- | --- |
|  | Women who delivered at home | | | | |  | Women who delivered in a facility | | | | |
|  | Time since delivery | CIF | SE | 95% CI | |  | Time since delivery | CIF | SE | 95% CI | |
|  |  |  |  | lower bound | upper bound |  |  |  |  | lower bound | upper bound |
| Women who had zero contacts where FP was discussed | <48 hrs | 0 |  |  |  |  | <48 hrs | 0 |  |  |  |
|  | <1 week | 0 |  |  |  |  | <1 week | 0 |  |  |  |
|  | 1 mo. | 0 |  |  |  |  | 1 mo. | 0.0128 | 0.0127 | 0.0011 | 0.0616 |
|  | 2 mo. | 0.0511 | 0.0166 | 0.0252 | 0.0907 |  | 2 mo. | 0.141 | 0.0394 | 0.075 | 0.2274 |
|  | 3 mo. | 0.0852 | 0.021 | 0.0499 | 0.1323 |  | 3 mo. | 0.2821 | 0.051 | 0.1875 | 0.3841 |
|  | 4 mo. | 0.1136 | 0.0239 | 0.0721 | 0.1655 |  | 4 mo. | 0.3077 | 0.0523 | 0.2094 | 0.4112 |
|  | 5 mo. | 0.125 | 0.0249 | 0.0813 | 0.1785 |  | 5 mo. | 0.3205 | 0.0528 | 0.2206 | 0.4246 |
|  | 6 mo. | 0.1534 | 0.0272 | 0.1048 | 0.2106 |  | 6 mo. | 0.3974 | 0.0554 | 0.2892 | 0.5035 |
|  | 7 mo. | 0.1648 | 0.028 | 0.1144 | 0.2233 |  | 7 mo. | no PPFP adoption | | | |
|  | 8 mo. | 0.1705 | 0.0283 | 0.1192 | 0.2296 |  | 8 mo. | 0.4103 | 0.0557 | 0.3009 | 0.5164 |
|  | 9 mo. | 0.2102 | 0.0307 | 0.1535 | 0.2731 |  | 9 mo. | 0.4487 | 0.0563 | 0.3365 | 0.5546 |
|  | 10 mo. | 0.2216 | 0.0313 | 0.1635 | 0.2854 |  | 10 mo. | no PPFP adoption | | | |
|  | 11 mo. | 0.2337 | 0.032 | 0.1741 | 0.2986 |  | 11 mo. | no PPFP adoption | | | |
|  | 12 mo. | 0.2413 | 0.0325 | 0.1805 | 0.3071 |  | 12 mo. | 0.4817 | 0.0576 | 0.3655 | 0.5885 |
|  | 13 mo. | 0.2961 | 0.0383 | 0.2234 | 0.3722 |  | 13 mo. | 0.5719 | 0.0625 | 0.4406 | 0.6832 |
|  |  |  |  |  |  |  |  |  |  |  |  |
| Women who had at least 1 contact where FP was discussed | <48 hrs | 0 |  |  |  |  | <48 hrs | 0.1111 | 0.0223 | 0.0722 | 0.1594 |
|  | <1 week | 0 |  |  |  |  | <1 week | 0.1162 | 0.0228 | 0.0763 | 0.1652 |
|  | 1 mo. | 0.0119 | 0.0068 | 0.0033 | 0.032 |  | 1 mo. | 0.1414 | 0.0248 | 0.0972 | 0.1937 |
|  | 2 mo. | 0.0909 | 0.0181 | 0.0595 | 0.1303 |  | 2 mo. | 0.3131 | 0.033 | 0.2499 | 0.3783 |
|  | 3 mo. | 0.1383 | 0.0217 | 0.0992 | 0.1839 |  | 3 mo. | 0.4091 | 0.0349 | 0.3403 | 0.4766 |
|  | 4 mo. | 0.1542 | 0.0227 | 0.1128 | 0.2014 |  | 4 mo. | 0.4343 | 0.0352 | 0.3646 | 0.502 |
|  | 5 mo. | 0.166 | 0.0234 | 0.1232 | 0.2144 |  | 5 mo. | 0.4646 | 0.0354 | 0.3939 | 0.5322 |
|  | 6 mo. | 0.2253 | 0.0263 | 0.176 | 0.2785 |  | 6 mo. | 0.5202 | 0.0355 | 0.4484 | 0.5871 |
|  | 7 mo. | 0.2372 | 0.0267 | 0.1868 | 0.2911 |  | 7 mo. | 0.5657 | 0.0352 | 0.4937 | 0.6313 |
|  | 8 mo. | 0.2609 | 0.0276 | 0.2085 | 0.3162 |  | 8 mo. | 0.601 | 0.0348 | 0.5293 | 0.6654 |
|  | 9 mo. | 0.2964 | 0.0287 | 0.2414 | 0.3534 |  | 9 mo. | 0.6313 | 0.0343 | 0.5601 | 0.6942 |
|  | 10 mo. | 0.3043 | 0.0289 | 0.2488 | 0.3616 |  | 10 mo. | 0.6364 | 0.0342 | 0.5652 | 0.699 |
|  | 11 mo. | 0.321 | 0.0294 | 0.2642 | 0.379 |  | 11 mo. | no PPFP adoption | | | |
|  | 12 mo. | 0.3469 | 0.0305 | 0.2877 | 0.4066 |  | 12 mo. | 0.643 | 0.0342 | 0.5717 | 0.7056 |
|  | 13 mo. | 0.3684 | 0.0319 | 0.3062 | 0.4306 |  | 13 mo. | 0.6522 | 0.0345 | 0.5799 | 0.7152 |

| **CIF of pregnancy** (stratified by place of delivery and whether woman reported at least 1 health system contact where FP was discussed) | | | | | | | | | | | |
| --- | --- | --- | --- | --- | --- | --- | --- | --- | --- | --- | --- |
| Women who had zero contacts where FP was discussed | 1 mo. | 0 |  |  |  |  | 1 mo. | 0.0385 | 0.0218 | 0.0103 | 0.0985 |
|  | 2 mo. | 0.0057 | 0.0057 | 0.0005 | 0.0289 |  | 2 mo. | no pregnancy | | | |
|  | 3 mo. | 0.0114 | 0.008 | 0.0023 | 0.0372 |  | 3 mo. | 0.0513 | 0.025 | 0.0166 | 0.116 |
|  | 4 mo. | 0.0341 | 0.0137 | 0.0141 | 0.0688 |  | 4 mo. | no pregnancy | | | |
|  | 5 mo. | no pregnancy | | | |  | 5 mo. | no pregnancy | | | |
|  | 6 mo. | 0.0455 | 0.0157 | 0.0213 | 0.0835 |  | 6 mo. | no pregnancy | | | |
|  | 7 mo. | 0.0511 | 0.0166 | 0.0252 | 0.0907 |  | 7 mo. | 0.0641 | 0.0277 | 0.0237 | 0.133 |
|  | 8 mo. | 0.0568 | 0.0174 | 0.0291 | 0.0978 |  | 8 mo. | 0.0769 | 0.0302 | 0.0314 | 0.1495 |
|  | 9 mo. | 0.0739 | 0.0197 | 0.0414 | 0.1187 |  | 9 mo. | 0.0897 | 0.0324 | 0.0395 | 0.1656 |
|  | 10 mo. | 0.0795 | 0.0204 | 0.0456 | 0.1255 |  | 10 mo. | no pregnancy | | | |
|  | 11 mo. | no pregnancy | | | |  | 11 mo. | no pregnancy | | | |
|  | 12 mo. | no pregnancy | | | |  | 12 mo. | no pregnancy | | | |
|  | 13 mo. | no pregnancy | | | |  | 13 mo. | no pregnancy | | | |
|  |  |  |  |  |  |  |  |  |  |  |  |
| Women who had at least 1 contact where FP was discussed | 1 mo. | 0.0119 | 0.0068 | 0.0033 | 0.032 |  | 1 mo. | 0.0051 | 0.005 | 0.0005 | 0.0259 |
|  | 2 mo. | 0.0158 | 0.0078 | 0.0053 | 0.0376 |  | 2 mo. | no pregnancy | | | |
|  | 3 mo. | no pregnancy | | | |  | 3 mo. | 0.0101 | 0.0071 | 0.002 | 0.0332 |
|  | 4 mo. | 0.0277 | 0.0103 | 0.0123 | 0.0536 |  | 4 mo. | no pregnancy | | | |
|  | 5 mo. | no pregnancy | | | |  | 5 mo. | no pregnancy | | | |
|  | 6 mo. | no pregnancy | | | |  | 6 mo. | 0.0152 | 0.0087 | 0.0042 | 0.0406 |
|  | 7 mo. | 0.0316 | 0.011 | 0.0149 | 0.0587 |  | 7 mo. | 0.0202 | 0.01 | 0.0067 | 0.0477 |
|  | 8 mo. | 0.0356 | 0.0116 | 0.0175 | 0.0638 |  | 8 mo. | no pregnancy | | | |
|  | 9 mo. | 0.0474 | 0.0134 | 0.0259 | 0.0786 |  | 9 mo. | no pregnancy | | | |
|  | 10 mo. | no pregnancy | | | |  | 10 mo. | no pregnancy | | | |
|  | 11 mo. | no pregnancy | | | |  | 11 mo. | 0.0255 | 0.0113 | 0.0096 | 0.0551 |
|  | 12 mo. | 0.0578 | 0.0151 | 0.033 | 0.0923 |  | 12 mo. | no pregnancy | | | |
|  | 13 mo. | no pregnancy | | | |  | 13 mo. | 0.0347 | 0.0144 | 0.0138 | 0.0715 |
|  | 14 mo. | 0.0721 | 0.0205 | 0.0387 | 0.1193 |  | 14 mo. | no pregnancy | | | |

**Table S2: MUltivariate competing-risks regression models to estimate the adjusted subhazards ratios of adoption of modern contraception over the first year postpartum for each type of mch contact**

| The adjusted subhazard ratio of adoption of modern contraception over the first year postpartum with pregnancy contacts with and without FP counseling | | | | | | | | |
| --- | --- | --- | --- | --- | --- | --- | --- | --- |
|  | **Home births** | | | | **Facility births** | | | |
|  |  |  | **95% CI** | |  |  | **95% CI** | |
|  | **aSHR** | **p-value** | **lower** | **upper** | **aSHR** | **p-value** | **lower** | **Upper** |
| **Pregnancy contacts** |  | | | | | | | |
| Contact with FP discussion | 1.30 | <0.001 | 1.16 | 1.44 | 1.14 | 0.001 | 1.05 | 1.23 |
| Contact without FP discussion | 0.97 | 0.683 | 0.86 | 1.10 | 1.05 | 0.243 | 0.97 | 1.15 |
| **Age of woman** |  |  |  |  |  |  |  |  |
| <=20 | 0.67 | 0.351 | 0.29 | 1.55 | 0.99 | 0.981 | 0.62 | 1.58 |
| 21-25 | reference group | | | | | | | |
| 26-30 | 1.17 | 0.449 | 0.78 | 1.74 | 0.76 | 0.126 | 0.53 | 1.08 |
| 31-35 | 0.95 | 0.885 | 0.47 | 1.92 | 0.55 | 0.083 | 0.28 | 1.08 |
| 36+ | 0.79 | 0.603 | 0.33 | 1.90 | 0.43 | 0.008 | 0.23 | 0.81 |
| **Marital status** |  | | | | | | | |
| Not married | reference group | | | | | | | |
| Married | 1.57 | 0.426 | 0.52 | 4.76 | 1.56 | 0.517 | 0.40 | 6.07 |
| **Years of education** |  | | | | | | | |
| None | reference group | | | | | | | |
| 1-8 years | 1.34 | 0.131 | 0.92 | 1.95 | 1.35 | 0.144 | 0.90 | 2.03 |
| 9+ years | 1.89 | 0.145 | 0.80 | 4.44 | 1.84 | 0.016 | 1.12 | 3.03 |
| **Religion** |  | | | | | | | |
| Orthodox | reference group | | | | | | | |
| Muslim | 0.28 | <0.001 | 0.19 | 0.41 | 0.65 | 0.020 | 0.45 | 0.93 |
| Other | 2.40 | 0.034 | 1.07 | 5.39 | 1.19 | 0.703 | 0.49 | 2.85 |
| **Wealth quintile** |  | | | | | | | |
| Lowest | reference group | | | | | | | |
| Second | 1.01 | 0.969 | 0.51 | 2.03 | 0.77 | 0.606 | 0.28 | 2.08 |
| Middle | 0.82 | 0.533 | 0.44 | 1.53 | 0.84 | 0.694 | 0.36 | 1.98 |
| Fourth | 0.85 | 0.624 | 0.44 | 1.63 | 1.19 | 0.670 | 0.53 | 2.70 |
| Highest | 0.96 | 0.908 | 0.44 | 2.06 | 1.06 | 0.901 | 0.45 | 2.50 |
| **Live children** |  | | | | | | | |
| 0 | reference group | | | | | | | |
| 1-2 | 1.99 | 0.502 | 0.27 | 14.97 | 1.69 | 0.376 | 0.53 | 5.40 |
| 3-4 | 1.38 | 0.748 | 0.19 | 9.79 | 1.83 | 0.317 | 0.56 | 6.00 |
| 5+ | 0.79 | 0.816 | 0.11 | 5.81 | 1.11 | 0.873 | 0.30 | 4.08 |
| **Study arm** |  | | | | | | | |
| Comparison arm | reference group | | | | | | | |
| Intervention arm | 1.15 | 0.462 | 0.79 | 1.69 | 0.86 | 0.353 | 0.63 | 1.18 |

| The adjusted subhazard ratio of adoption of modern contraception over the first year postpartum with and without FP counseling before discharge following birth | | | | | |
| --- | --- | --- | --- | --- | --- |
|  | **Home births** | **Facility births** | | | |
|  |  |  |  | **95% CI** | |
|  |  | **aSHR** | **p-value** | **lower** | **upper** |
| **Delivery contact** |  |  | | | |
| FP discussed pre-discharge |  | 1.76 | <0.001 | 1.32 | 2.35 |
| FP not discussed pre-discharge |  | reference group | | | |
| **Age of woman** |  |  | | | |
| <=20 |  | 1.05 | 0.838 | 0.65 | 1.69 |
| 21-25 |  | reference group | | | |
| 26-30 |  | 0.80 | 0.226 | 0.55 | 1.15 |
| 31-35 |  | 0.61 | 0.149 | 0.31 | 1.19 |
| 36+ |  | 0.50 | 0.041 | 0.26 | 0.97 |
| **Marital status** |  |  | | | |
| Not married |  | reference group | | | |
| Married |  | 1.49 | 0.562 | 0.39 | 5.70 |
| **Years of education** |  |  | | | |
| None |  | reference group | | | |
| 1-8 years |  | 1.28 | 0.271 | 0.82 | 2.01 |
| 9+ years |  | 1.82 | 0.025 | 1.08 | 3.07 |
| **Religion** |  |  | | | |
| Orthodox |  | reference group | | | |
| Muslim |  | 0.65 | 0.025 | 0.44 | 0.95 |
| Other |  | 0.87 | 0.779 | 0.34 | 2.27 |
| **Wealth quintile** |  |  | | | |
| Lowest |  | reference group | | | |
| Second |  | 0.83 | 0.713 | 0.30 | 2.27 |
| Middle |  | 0.91 | 0.828 | 0.40 | 2.09 |
| Fourth |  | 1.16 | 0.721 | 0.52 | 2.57 |
| Highest |  | 1.10 | 0.816 | 0.48 | 2.56 |
| **Live children** |  |  | | | |
| 0 |  | reference group | | | |
| 1-2 |  | 1.81 | 0.326 | 0.55 | 5.92 |
| 3-4 |  | 1.86 | 0.312 | 0.56 | 6.18 |
| 5+ |  | 1.06 | 0.927 | 0.28 | 4.00 |
| **Study arm** |  |  | | | |
| Comparison arm |  | reference group | | | |
| Intervention arm |  | 0.93 | 0.673 | 0.67 | 1.29 |

| The adjusted subhazard ratio of adoption of modern contraception over the first year postpartum with PNC contacts with and without FP counseling | | | | | | | | | |
| --- | --- | --- | --- | --- | --- | --- | --- | --- | --- |
|  | **Home births** | | | | **Facility births** | | | |  |
|  |  |  | **95% CI** | |  |  | **95% CI** | |  |
|  | **aSHR** | **p-value** | **lower** | **upper** | **aSHR** | **p-value** | **lower** | **upper** |  |
| **PNC contacts** |  |  |  |  |  |  |  |  |  |
| Contact with FP discussion | 1.08 | 0.298 | 0.94 | 1.24 | 1.15 | <0.001 | 1.07 | 1.24 |  |
| Contact without FP discussion | 1.13 | 0.050 | 1.00 | 1.28 | 0.95 | 0.296 | 0.87 | 1.04 |  |
| **Age of woman** |  |  |  |  |  |  |  |  |  |
| <=20 | 0.61 | 0.231 | 0.27 | 1.37 | 0.95 | 0.819 | 0.59 | 1.52 |  |
| 21-25 | reference group | | | | | | | |  |
| 26-30 | 1.13 | 0.523 | 0.77 | 1.67 | 0.72 | 0.055 | 0.51 | 1.01 |  |
| 31-35 | 0.82 | 0.593 | 0.40 | 1.68 | 0.54 | 0.075 | 0.27 | 1.06 |  |
| 36+ | 0.80 | 0.618 | 0.33 | 1.93 | 0.42 | 0.008 | 0.22 | 0.80 |  |
| **Marital status** |  | | | | | | | |  |
| Not married | reference group | | | | | | | |  |
| Married | 1.24 | 0.758 | 0.32 | 4.85 | 1.54 | 0.536 | 0.39 | 6.13 |  |
| **Years of education** |  | | | | | | | |  |
| None | reference group | | | | | | | |  |
| 1-8 years | 1.21 | 0.305 | 0.84 | 1.75 | 1.31 | 0.217 | 0.85 | 2.03 |  |
| 9+ years | 1.67 | 0.282 | 0.66 | 4.26 | 1.99 | 0.007 | 1.21 | 3.29 |  |
| **Religion** |  | | | | | | | |  |
| Orthodox | reference group | | | | | | | |  |
| Muslim | 0.31 | <0.001 | 0.20 | 0.47 | 0.61 | 0.009 | 0.42 | 0.88 |  |
| Other | 3.18 | 0.010 | 1.32 | 7.66 | 1.06 | 0.898 | 0.43 | 2.58 |  |
| **Wealth quintile** |  | | | | | | | |  |
| Lowest | reference group | | | | | | | |  |
| Second | 1.03 | 0.934 | 0.50 | 2.11 | 0.85 | 0.751 | 0.30 | 2.37 |  |
| Middle | 0.87 | 0.680 | 0.46 | 1.66 | 0.83 | 0.685 | 0.34 | 2.02 |  |
| Fourth | 0.88 | 0.726 | 0.44 | 1.77 | 1.15 | 0.741 | 0.50 | 2.66 |  |
| Highest | 0.87 | 0.748 | 0.38 | 1.99 | 1.03 | 0.946 | 0.43 | 2.49 |  |
| **Live children** |  | | | | | | | |  |
| 0 | reference group | | | | | | | |  |
| 1-2 | 1.62 | 0.652 | 0.20 | 13.13 | 1.85 | 0.305 | 0.57 | 6.00 |  |
| 3-4 | 1.22 | 0.848 | 0.16 | 9.19 | 2.10 | 0.226 | 0.63 | 6.94 |  |
| 5+ | 0.80 | 0.827 | 0.11 | 5.78 | 1.24 | 0.745 | 0.34 | 4.59 |  |
| **Study arm** |  | | | | | | | |  |
| Comparison arm | reference group | | | | | | | |  |
| Intervention arm | 1.41 | 0.076 | 0.96 | 2.06 | 0.92 | 0.582 | 0.67 | 1.26 |  |

| The adjusted subhazard ratio of adoption of modern contraception over the first year postpartum with immunization contacts with and without FP counseling | | | | | | | | |
| --- | --- | --- | --- | --- | --- | --- | --- | --- |
|  | **Home births** | | | | **Facility births** | | | |
|  |  |  | **95% CI** | |  |  | **95% CI** | |
|  | **aSHR** | **p-value** | **lower** | **upper** | **aSHR** | **p-value** | **lower** | **upper** |
| **Immunization contacts** |  | | | | | | | |
| Contact with FP discussion | 1.36 | <0.001 | 1.17 | 1.57 | 1.17 | 0.040 | 1.01 | 1.36 |
| Contact without FP discussion | 1.18 | 0.006 | 1.05 | 1.33 | 1.02 | 0.776 | 0.89 | 1.17 |
| **Age of woman** |  |  |  |  |  |  |  |  |
| <=20 | 0.60 | 0.234 | 0.26 | 1.39 | 0.99 | 0.957 | 0.60 | 1.61 |
| 21-25 | reference group | | | | | | | |
| 26-30 | 1.19 | 0.379 | 0.81 | 1.76 | 0.75 | 0.077 | 0.54 | 1.03 |
| 31-35 | 0.91 | 0.777 | 0.46 | 1.80 | 0.54 | 0.084 | 0.27 | 1.09 |
| 36+ | 0.87 | 0.756 | 0.35 | 2.14 | 0.42 | 0.005 | 0.23 | 0.77 |
| **Marital status** |  | | | | | | | |
| Not married | reference group | | | | | | | |
| Married | 1.23 | 0.724 | 0.40 | 3.81 | 1.31 | 0.693 | 0.34 | 5.03 |
| **Years of education** |  |  |  |  |  |  |  |  |
| None | reference group | | | | | | | |
| 1-8 years | 1.18 | 0.386 | 0.81 | 1.70 | 1.30 | 0.213 | 0.86 | 1.98 |
| 9+ years | 1.28 | 0.634 | 0.47 | 3.46 | 1.86 | 0.018 | 1.11 | 3.10 |
| **Religion** |  | | | | | | | |
| Orthodox | reference group | | | | | | | |
| Muslim | 0.32 | <0.001 | 0.21 | 0.48 | 0.58 | 0.004 | 0.40 | 0.84 |
| Other | 1.72 | 0.240 | 0.70 | 4.23 | 0.96 | 0.930 | 0.40 | 2.30 |
| **Wealth quintile** |  | | | | | | | |
| Lowest | reference group | | | | | | | |
| Second | 0.94 | 0.858 | 0.46 | 1.89 | 0.79 | 0.656 | 0.28 | 2.22 |
| Middle | 0.84 | 0.593 | 0.45 | 1.57 | 0.82 | 0.652 | 0.34 | 1.97 |
| Fourth | 0.78 | 0.490 | 0.39 | 1.56 | 1.21 | 0.652 | 0.53 | 2.77 |
| Highest | 1.06 | 0.890 | 0.49 | 2.29 | 1.05 | 0.905 | 0.44 | 2.51 |
| **Live children** |  | | | | | | | |
| 0 | reference group | | | | | | | |
| 1-2 | 1.11 | 0.927 | 0.13 | 9.33 | 1.70 | 0.411 | 0.48 | 6.07 |
| 3-4 | 0.83 | 0.862 | 0.11 | 6.53 | 1.74 | 0.406 | 0.47 | 6.47 |
| 5+ | 0.49 | 0.489 | 0.07 | 3.66 | 1.06 | 0.932 | 0.25 | 4.45 |
| **Study arm** |  | | | | | | | |
| Comparison arm | reference group | | | | | | | |
| Intervention arm | 1.30 | 0.189 | 0.88 | 1.91 | 0.89 | 0.480 | 0.65 | 1.23 |
