# Supplementary material for: Effect of multiple counselling contacts along the continuum of care on use of postpartum family planning in a cohort of Ethiopian women: a dose-response analysis
Source: BMJ Open. 2024 Dec 20;14(12):e084247. doi: 10.1136/bmjopen-2024-084247 (PMC11667413; doi:10.1136/bmjopen-2024-084247)
Supplement: Supplementary file 2 [file bmjopen-2024-084247.reviewer_comments.pdf]

PEER REVIEW HISTORY

BMJ Open publishes all reviews undertaken for accepted manuscripts. Reviewers are asked to complete a checklist review form and are provided with free text boxes to elaborate on their assessment. These free text comments are reproduced below.

ARTICLE DETAILS

Title (Provisional)

A dose response analysis of the effect of multiple counseling contacts along the continuum of care on use of postpartum family planning in a cohort of Ethiopian women

Authors

Pfitzer, Anne; Jima, Gebi Husein; Sitrin, Deborah; Ayalew, Firew; Ahmed, Saifuddin

---

VERSION 1 - REVIEW

**Reviewer**	**1**
**Name**	**Balcha, Wondu**
**Affiliation**	**Bahir Dar University College of Medical and Health Sciences, Midwifery**
**Date**	**01-Feb-2024**
**COI**	**I have no competing interests**

- **Title:** A DOSE RESPONSE ANALYSIS OF THE EFFECT OF MULTIPLE COUNSELING CONTACTS ALONG THE CONTINUUM OF CARE ON USE OF POSTPARTUM FAMILY PLANNING IN A COHORT OF ETHIOPIAN WOMEN

Comment: it is best if you make it extended postpartum period

- Abstract –it has been written well. Avoid before using the abbreviation write it first in full words (e.g., see line number 32) and use them consistently. If you start using the abbreviations or acronyms once, you don't have write the full words thereafter (see postpartum family planning and PPFP) –revise it
- Strength and limitation of the study – it is good. However, in your study sitting the proportion of home delivery was higher. Your study was cohort or interventional study and starting from the first interview your data collectors was health care providers including health extension workers. So did you counseled them to give childbirth at health facility or not. If not it was also your study limitation, because the figure is higher.

- Generally comment – you need to be consistent thought your document. For example you said 4-9 months in the main manuscript document (see line number 142) and again 6-9 months in your supplementary material –see it
- Methods – some of the methods described, but you miss to include some of the methods section. Just like inclusion and exclusion criteria. I saw it in the supplementary material section, try to include it in methods section. How did you excluded non-pregnant women, because they are not your study population? The exclusion must be from the inclusion.
- Result and discussion – the result and discussion of the study written and explained soundly.
- Generally comment – you need to be consistent thought your document. For example you said 4-9 months in the main manuscript document (see line number 142) and again 6-9 months in your supplementary material –see it.

**Reviewer**	**2**
**Name**	**Ujah, O.I.**
**Affiliation**	**University of South Florida**
**Date**	**05-Jul-2024**
**COI**	**N/A**

This manuscript presents a dose-response analysis to evaluate the impact of each additional antenatal, delivery, postnatal, or child immunization contact, where family planning (FP) was discussed, on the uptake of postpartum family planning (PPFP) up to 12 months postpartum from a Sub-Saharan African population. The article is well written and makes meaningful contribution to the scarce literature.

**Reviewer**	**3**
**Name**	**Saansong , Ahmed**
**Affiliation<br>and Education**	**University of Education Winneba, Health Administration**
**Date**	**17-Aug-2024**
**COI**	**None.**

General comments

Strengths

1.The presented abstract effectively summarizes the study's aims, methods, and key findings, serving as a clear roadmap for readers.

2.The methodology outlined is comprehensive. It details the study design, setting, interventions, and sample size, enhancing the reader's understanding of the research process and the reliability of the study's findings.

3.Ethical concerns are well addressed, particularly regarding the consent process for low-literate participants. This demonstrates a commitment to ethical sensitivity and informed consent on the part of the authors of this study. Kudos.

4.The data is presented in a thorough manner, with clear operational definitions and statistical analyses, including adjusted subhazard ratios (aSHRs), confidence intervals, and p-values, which bolster this study's internal validity.

Weaknesses

1.The reliance on women's recall of contraception adoption introduces bias, yet the paper lacks substantial strategies to mitigate this issue, such as data triangulation or sensitivity analysis. Cross-verification with medical records could have improved reliability.

2.While some limitations, such as bias and generalisability issues, are acknowledged, they are not explored in depth. For instance, there should be a paragraph/few lines in the methodology addressing the implications of the rapid qualitative assessment for selecting study areas should be elaborated to better understand its impact on the study's external validity.

3.Although sophisticated statistical methods are employed, the paper does not provide a clear rationale for their selection. An explanation of why life table analysis and multivariate analysis are suitable for this dataset would enhance the findings' robustness.

4.This study claims to generalise findings to a larger population but fails to substantiate this adequately. Detailed information about the representativeness of the sample would reinforce this assertion.

5.The authors occasionally lack coherence in presenting their arguments. There should be clear linking statements between sections and explicit statements of the research's hypothesis. This would strengthen the overall argument structure.

6.While the main findings and their significance are reiterated, the authors do not thoroughly explore broader implications these findings. They could discuss the integration of postpartum family planning (PPFP) services in various healthcare settings and potential challenges.

7.Although the authors advocate for the systematic integration of PPFP services, they failed to present concrete examples and actionable recommendations for policymakers and healthcare providers. More detailed policy suggestions or case studies would enhance practical application of this study's findings.

Specific Comments

General presentation

1.The authors employ specialised terms such as "dose-response analysis" and "competitive-risk regression" without sufficient clarification for readers who may not be familiar with these concepts. It would be beneficial to provide clear definitions or footnotes on such terms to enhance comprehension. You explaining that a dose-response analysis evaluates how varying levels of exposure—such as counseling contacts—impact outcomes like postpartum family planning (PPFP) uptake would significantly aid understanding for audience.

2.Acronyms and technical terms are frequently used without explanations, which can confuse readers unfamiliar with the terms. Provide a glossary or define terms upon first use to enhance clarity and understanding. For instance, instead of repeatedly using "PPFP" without explanation, the first instance should read "Postpartum Family Planning (PPFP)."

3.The transitions between sections, particularly from intervention arms to dose-response analysis, are abrupt and lack the necessary bridges. A smoother transition could enhance readability and coherence. After discussing the multiple counseling contacts, you can use an introductory sentence such as, "To quantitatively assess the effectiveness, we conducted a dose-response analysis to measure the relative impact of varying levels of counseling," would provide a clearer pathway for readers

4.While the methodology is detailed, presenting it in a more concise and clear manner would greatly benefit the reader. I struggled to grasp the various aspects and concepts used of your methodology. Please breakdown the methodology and analysis into manageable steps with subheadings such as research design, setting, population, sampling technique and size etc. and bullet points to simplify procedures. You can use a table to summarise the key analysis steps and their purposes to make this information more digestible and user-friendly. The methods section is overly complex and could alienate readers who are not experts in this field.

5.The authors need to incorporate real-world examples or case studies to enhance this paper's relatability. For instance, they could describe a typical encounter during a maternal health visit where family planning is discussed can provide tangible context. An example could be, "During routine antenatal visits, health workers provided counseling on various contraceptive methods, emphasizing the benefits of PPFP for child spacing and maternal health." Such illustrations will bridge the gap between theory and practice.

6.The authors mention limitations such as potential recall bias and underreporting, however, they did not adequately explore the implications of these limitations to the study’s finding. This section needs to be reviewed and made substantial. A critical examination of how these limitations might influence the study's validity would provide a more balanced perspective. An example could be, "Potential recall bias may have led to over- or under-reporting of

counseling contacts, which could affect the study's estimates of the impact on PPFP uptake. Acknowledging these limitations emphasize the importance of corroborating self-reported data with health records."

7.The paper concludes without summarizing the main study findings or discussing their broader implications. Please revise the conclusion section. I understand trying to beat the word count required by the journal but this should not affect key sections of the paper. A well-rounded conclusion which encompasses the main objective, the findings, recommendations for both policy and future studies is needed to reinforce the study's significance and open avenues for further inquiry.

Areas needing additional sources and citations:

1.The paper lacks a strong introductory statement that outlines the significance of the study and the research gap it aims to fill. The authors need to reference existing literature that discusses the current challenges in PPFP in Ethiopia and highlight how their study addresses these issues. For instance, if the study claims that there is limited integration of family planning services into maternal, newborn, and child health (MNCH) contacts, it should reference studies or reports that support this statement.

2.While the use of household surveys is a strength, the authors need to provide a detailed reference to the methodologies employed to ensure the representativeness of the sample. Citing methodologies from established surveys, like the Demographic and Health Surveys (DHS), would enhance the credibility of the findings. For example, stating that "the representativeness of the sample was ensured by adopting methodologies similar to those used in large-scale surveys like the DHS" would be beneficial. (Rutstein & Johnson, 2004)

3.To support the credibility of this study’afindings, the authors should include citations for the statistical methods used. Discussing the appropriateness of these methods in the context of the study design is essential. For instance, referencing a study like Fitzmaurice et al. (2012) on multivariate competing-risks regression models would provide rigor to the statistical analysis employed in the study. "Fitzmaurice, G. M., Laird, N. M., & Ware, J. H. (2012). Applied Longitudinal Analysis. John Wiley & Sons."

4.Certain claims, such as the impact of postnatal PPFP counseling on uptake, require additional support from referenced studies. The authors should include citations from previous research that corroborates their findings regarding the effectiveness of counseling. For example, stating that "previous studies have demonstrated that postnatal counseling significantly increases the uptake of postpartum family planning (Smith et al., 2016; Jones et al., 2018)."" would strengthen this section.

5.The authors should provide comprehensive details about the study interventions, including the content and methods of family planning counseling. Incorporating specific examples or case studies from similar settings would enhance the understanding of how these interventions were implemented. Referencing guidelines, such as the WHO Family Planning

Guidance, would also add depth to this section. WHO Family Planning Guidance: https://www.who.int/reproductivehealth/publications/family_planning/en/

6.While ethical considerations are mentioned, a more in-depth discussion on potential challenges encountered during the study period is necessary. Citing ethical guidelines, such as those outlined in the Belmont Report (National Commission for the Protection of Human Subjects, 1979), would add credibility to the ethical framework of this study.

VERSION 1 - AUTHOR RESPONSE

Thank you for the continued collaboration on improving this paper. Per your last communication, I have reformatted the tables to make them less than 8 columns, and have also removed lines so that they are formatted more in the style of your journal. I have also de-linked the references and changed the numbering to use brackets [ ]. Lastly, we re-generated the figures using a more recent version of STATA to make the formatting a bit more presentable. I didn't reformat the supplemental materials however.

Response to Reviewer 1 comments

S.no	Reviewer 1 comments	Authors' response to the comments
1	Title: A DOSE RESPONSE ANALYSIS OF THE EFFECT OF MULTIPLE COUNSELING CONTACTS ALONG THE CONTINUUM OF CARE ON USE OF POSTPARTUM FAMILY PLANNING IN A COHORT OF ETHIOPIAN WOMEN<br>Comment: it is best if you make it extended postpartum period	WHO defines Postpartum Family Planning to refer to the use in the 12 months postpartum (see citation reference No 5 in our paper). Therefore, we do not think we need to make the title even longer than it already is. The text provides the clarity desired.
2	Abstract –it has been written well. Avoid before using the abbreviation write it first in full words (e.g., see line number 32) and use them consistently. If you start using the abbreviations or acronyms once, you don't have write the full words thereafter (see postpartum family planning and PPFP) –revise it	Thank you for pointing out this instance of the abbreviation. We corrected this.

S.no	Reviewer 1 comments	Authors' response to the comments
3	Strength and limitation of the study – it is good. However, in your study sitting the proportion of home delivery was higher. Your study was cohort or interventional study and starting from the first interview your data collectors was health care providers including health extension workers. So did you counseled them to give childbirth at health facility or not. If not it was also your study limitation, because the figure is higher.	The study was embedded into the real world health system. It is routine for antenatal care to include recommendations to deliver in a facility, so our interviews did not specifically ask about exposure to this recommendation. Women and their families make decisions about where to deliver based on a number of factors that go beyond the objectives of this study.
4	Generally comment – you need to be consistent thought your document. For example you said 4-9 months in the main manuscript document (see line number 142) and again 6-9 months in your supplementary material –see it	We believe the reviewer is referring to the protocol that was included per the journal guideline. In the protocol, we had not anticipated that women would disclose a pregnancy before 6 months. We removed that descriptor of 2nd and 3rd trimester as it is not material to our results.<br>We also note that women are not always able to accurately self-report the gestational age of their pregnancy, so the accuracy of these data may be questioned.
5	Methods – some of the methods described, but you miss to include some of the methods section. Just like inclusion and exclusion criteria. I saw it in the supplementary material section, try to include it in methods section. How did you excluded non-pregnant women, because they are not your study population? The exclusion must be from the inclusion.	The inclusion criteria was any pregnant women and is described in lines 140-142. Given the house-to-house identification of pregnant women, it would be difficult to identify women in their first trimester of pregnancy, so they were excluded de-facto rather than by explicit exclusion.
6	Result and discussion – the result and discussion of the study written and explained soundly.	Thank you for this positive comment.
7	Generally comment – you need to be consistent thought your document. For example you said 4-9 months in the main manuscript document (see line number 142) and again 6-9 months in your supplementary material –see it.	See response to comment 4 above. This seems to be a duplicate point.

Response to Reviewer 2 comments

**S.no**	**Reviewer 2 comments**	**Authors' response to the comments**
1	This manuscript presents a dose-response analysis to evaluate the impact of each additional antenatal, delivery, postnatal, or child immunization contact, where family planning (FP) was discussed, on the uptake of [illegible]	Thank you

Response to Reviewer 3 comments

S.no	**Reviewer 3 comments**	**Authors' response to the comments**
1	The presented abstract effectively summarizes the study's aims, methods, and key findings, serving as a clear roadmap for readers.	Thank you for this observation. We appreciate your thorough review of our paper and taking the time to highlight its strengths along with making your constructive recommendations.
2	The methodology outlined is comprehensive. It details the study design, setting, interventions, and sample size, enhancing the reader's understanding of the research process and the reliability of the study's findings.	Thank you for taking the time to make this point.
3	Ethical concerns are well addressed, particularly regarding the consent process for low-literate participants. This demonstrates a commitment to ethical sensitivity and informed consent on the part of the authors of this study. Kudos.	Thank you.

S.no	Reviewer 3 comments	Authors' response to the comments
4	The data is presented in a thorough manner, with clear operational definitions and statistical analyses, including adjusted subhazard ratios (aSHRs), confidence intervals, and p-values, which bolster this study's internal validity.	Thank you
1	The reliance on women's recall of contraception adoption introduces bias, yet the paper lacks substantial strategies to mitigate this issue, such as data triangulation or sensitivity analysis. Cross-verification with medical records could have improved reliability.	The reviewer points to a limitation in our methods that rely on self-report for data on contacts. Data triangulation would not have been feasible in the context due to privacy and logistical concerns related to the nature of health records in health posts and health centers. Furthermore, we identified 28 women who didn't have any visits to health facilities and thus have no facility records (see lines 419-420). We carefully thought through the methodology for asking questions to improve the recall, such as tying the recall to a specific point of service.<br>As for sensitivity analysis, we did run analyses not presented here, which did not result in meaningful difference.
2	While some limitations, such as bias and generalisability issues, are acknowledged, they are not explored in depth. For instance, there should be a paragraph/few lines in the methodology addressing the implications of the rapid qualitative assessment for selecting study areas should be elaborated to better understand its impact on the study's external validity.	The rapid qualitative assessment was carried out in different woreda than the study, so we don't believe there are implications of that exercise on our results or their generalizability, and have added a sentence as to why that is.
3	Although sophisticated statistical methods are employed, the paper does not provide a clear rationale for their selection. An explanation of why life table analysis and multivariate analysis are suitable for this dataset would enhance the findings' robustness.	We modified the section on life table analysis to explain the rationale for this statistical method.<br>We left it as is for the multivariate analysis, as the paper in lines 170-172 does explain that "we conducted multivariate analysis to adjust for the effect of potential confounding covariates on PPFP adoption hazard rates. "

S.no	Reviewer 3 comments	Authors' response to the comments
4	This study claims to generalise findings to a larger population but fails to substantiate this adequately. Detailed information about the representativeness of the sample would reinforce this assertion.	The paper described briefly how community guides and health extension workers helped us identify pregnant women (line 140), where they even identified 28 women who had zero contacts with the formal health system (419-420). While we can't guarantee that no pregnant women were missed at community level, the numbers included in our sample and the selection from the community, rather than the health system, gives us a sense that we are representing women who include those infrequent users of health services. However, we have removed the generalizability claim as unnecessary.
5	The authors occasionally lack coherence in presenting their arguments. There should be clear linking statements between sections and explicit statements of the research's hypothesis. This would strengthen the overall argument structure.	We dispute that the research misses a clear hypothesis, as we feel this is included in lines 100-103 of the paper. And the research question is stated in lines 113-115. While we do acknowledge that this analysis is complex, we have sought to bring back the focus on the question in the discussion section.
6	While the main findings and their significance are reiterated, the authors do not thoroughly explore broader implications these findings. They could discuss the integration of postpartum family planning (PPFP) services in various healthcare settings and potential challenges.	It is beyond the scope of this particular paper to discuss the challenges of integration, however we have added a sentence to point readers to our other paper on the qualitative results of this study in the Discussion (line 387).
7	Although the authors advocate for the systematic integration of PPFP services, they failed to present concrete examples and actionable recommendations for policymakers and healthcare providers. More detailed policy suggestions or case studies would enhance practical application of this study's findings.	As noted above, such a discussion is beyond the scope of this paper; however, we published another, qualitative paper linked to this study, which does offer such recommendations. However, this paper provides modeled estimates of what success would generated in terms of PPFP use among postpartum women.

S.no	Reviewer 3 comments	Authors' response to the comments
1	The authors employ specialised terms such as "dose-response analysis" and "competitive-risk regression" without sufficient clarification for readers who may not be familiar with these concepts. It would be beneficial to provide clear definitions or footnotes on such terms to enhance comprehension. You explaining that a dose-response analysis evaluates how varying levels of exposure—such as counseling contacts—impact outcomes like postpartum family planning (PPFP) uptake would significantly aid understanding for audience.	Related to terminology we used, see lines 177-193 where we provide a detailed definition of a dose, and what questions and how they were asked to arrive at a dose in terms of MNCH contracts with the health system and whether or not these contacts included a discussion of FP. We also feel that the implication is clear that the response is the adoption of PPFP. Related to competitive risk regression, this terminology is explained in lines 207-211 as well as in the statistical methods references offered in citation. We are not aware of BMJ Open encouraging the use of explanatory footnotes in its guidance to authors.
2	Acronyms and technical terms are frequently used without explanations, which can confuse readers unfamiliar with the terms. Provide a glossary or define terms upon first use to enhance clarity and understanding. For instance, instead of repeatedly using "PPFP" without explanation, the first instance should read "Postpartum Family Planning (PPFP)."	We are not aware of BMJ Open using glossaries but we appreciate the recommendation to spell out abbreviations at first use, which we have attempted to do.
3	The transitions between sections, particularly from intervention arms to dose-response analysis, are abrupt and lack the necessary bridges. A smoother transition could enhance readability and coherence. After discussing the multiple counseling contacts, you can use an introductory sentence such as, "To quantitatively assess the effectiveness, we conducted a dose-response analysis to measure the relative impact of varying levels of counseling," would provide a clearer pathway for readers	Thank you for this suggestion. We have added such a transition in line 202.

S.no	**Reviewer 3 comments**	**Authors' response to the comments**
4	While the methodology is detailed, presenting it in a more concise and clear manner would greatly benefit the reader. I struggled to grasp the various aspects and concepts used of your methodology. Please breakdown the methodology and analysis into manageable steps with subheadings such as research design, setting, population, sampling technique and size etc. and bullet points to simplify procedures. You can use a table to summarise the key analysis steps and their purposes to make this information more digestible and user-friendly. The methods section is overly complex and could alienate readers who are not experts in this field.	Thank you for the suggestion. We have added sub-headings to describe the different analyses carried out. We already had to justify the number of tables and figures to the editors as we are over their recommended limit, so we omitted adding another table.
5	The authors need to incorporate real-world examples or case studies to enhance this paper's relatability. For instance, they could describe a typical encounter during a maternal health visit where family planning is discussed can provide tangible context. An example could be, "During routine antenatal visits, health workers provided counseling on various contraceptive methods, emphasizing the benefits of PPFP for child spacing and maternal health." Such illustrations will bridge the gap between theory and practice.	We appreciate this recommendation, but feel it is not appropriate for the type of article this represents. We do encourage the reviewer to read our qualitative study paper which provides rich description of interactions between providers and clients.

S.no	Reviewer 3 comments	Authors' response to the comments
6	The authors mention limitations such as potential recall bias and underreporting, however, they did not adequately explore the implications of these limitations to the study's finding. This section needs to be reviewed and made substantial. A critical examination of how these limitations might influence the study's validity would provide a more balanced perspective. An example could be, "Potential recall bias may have led to over- or under-reporting of counseling contacts, which could affect the study's estimates of the impact on PPFP uptake. Acknowledging these limitations emphasize the importance of corroborating self-reported data with health records."	We believe we have enumerated the limitations, including those that may lead to under-estimating the effects. See line 429 for example.
7	The paper concludes without summarizing the main study findings or discussing their broader implications. Please revise the conclusion section. I understand trying to beat the word count required by the journal but this should not affect key sections of the paper. A well-rounded conclusion which encompasses the main objective, the findings, recommendations for both policy and future studies is needed to reinforce the study's significance and open avenues for further inquiry.	The first paragraph of the discussion section (specifically lines 335-338) does indeed summarize the main finding of a dose-response effect of FP discussions on use. The subsequent paragraph describes the evidence for timing or type of contact where FP can be integrated. The third paragraph discusses implications of low integration found in our study and how global efforts seeking to improve measurement of integration seek to address these gaps. The fourth paragraph shares the result of modeling estimates if integration were to be achieved across 8 contacts, which we feel is realistic considering expectations for antenatal, intrapartum and postnatal and extended postpartum visits. In short, we are not sure what the reviewer means with this comment.

S.no	**Reviewer 3 comments**	**Authors' response to the comments**
1	The paper lacks a strong introductory statement that outlines the significance of the study and the research gap it aims to fill. The authors need to reference existing literature that discusses the current challenges in PPFP in Ethiopia and highlight how their study addresses these issues. For instance, if the study claims that there is limited integration of family planning services into maternal, newborn, and child health (MNCH) contacts, it should reference studies or reports that support this statement.	Our paper indicates in lines 100-103 what the gap in the current evidence about integration, namely that existing literature does not link evidence of integration in ANC and at births, and that of integration during immunization visits into a holistic view of what a health system can achieve to meet postpartum women's needs for healthy timing and spacing of pregnancies. Furthermore, we have cited the literature extensively.
2	While the use of household surveys is a strength, the authors need to provide a detailed reference to the methodologies employed to ensure the representativeness of the sample. Citing methodologies from established surveys, like the Demographic and Health Surveys (DHS), would enhance the credibility of the findings. For example, stating that "the representativeness of the sample was ensured by adopting methodologies similar to those used in large-scale surveys like the DHS" would be beneficial. (Rutstein & Johnson, 2004)	We appreciate the concern with the strength of household survey. However, our study team did not aspire to the rigor espoused by the DHS, even if we did utilize some questions from their questionnaires. We thus cannot exaggerate. However, we did conduct a statistical power calculation to ensure that our sample could answer our research question.
3	To support the credibility of this study'a findings, the authors should include citations for the statistical methods used. Discussing the appropriateness of these methods in the context of the study design is essential. For instance, referencing a study like Fitzmaurice et al. (2012) on multivariate competing-risks regression models would provide rigor to the statistical analysis employed in the study. "Fitzmaurice, G. M., Laird, N. M., & Ware, J. H. (2012). Applied Longitudinal Analysis. John Wiley & Sons."	We believe we have done so with citations 16,17,18,19.

S.no	Reviewer 3 comments	Authors' response to the comments
4	Certain claims, such as the impact of postnatal PPFP counseling on uptake, require additional support from referenced studies. The authors should include citations from previous research that corroborates their findings regarding the effectiveness of counseling. For example, stating that "previous studies have demonstrated that postnatal counseling significantly increases the uptake of postpartum family planning (Smith et al., 2016; Jones et al., 2018)."" would strengthen this section.	We had a discussion of the literature in the introduction (see lines 71-83) and another lengthy paragraph in the discussion starting on line 407 that includes articles such as the one by Zimmerman et al looking at postnatal counseling in Ethiopia among other contacts. However, there have been additional papers published since we started writing this manuscript. We have added a citation from Nepal to that point in that paragraph in the discussion.
5	The authors should provide comprehensive details about the study interventions, including the content and methods of family planning counseling. Incorporating specific examples or case studies from similar settings would enhance the understanding of how these interventions were implemented. Referencing guidelines, such as the WHO Family Planning Guidance, would also add depth to this section. WHO Family Planning Guidance: https://www.who.int/reproductivehealth/publications/family_planning/en/	Thank you for this suggestion; however, we are also careful not to repeat descriptions already published in other papers related to this study and which are cited. We have included the WHO guidance as citation number 5.
6	While ethical considerations are mentioned, a more in-depth discussion on potential challenges encountered during the study period is necessary. Citing ethical guidelines, such as those outlined in the Belmont Report (National Commission for the Protection of Human Subjects, 1979), would add credibility to the ethical framework of this study.	Our study maintained close adherence to ethical requirements, despite this being a low-risk study, with frequent reporting to the IRB at JHU. We don't feel that we've deviated from reporting on ethical guidelines in this paper.

VERSION 2 - REVIEW

Reviewer 1

Name Balcha, Wondu

Affiliation Bahir Dar University College of Medical and Health Sciences, Midwifery

Date 21-Nov-2024

COI

The comments and suggestions have been addressed well.
